# Comprehensive transcriptomic analysis identifies SLC25A4 as a key predictor of prognosis in osteosarcoma

**DOI:** 10.3389/fgene.2024.1410145

**Published:** 2024-06-18

**Authors:** Ying Zhang, Yinghui Wang, Wenyan Zhang, Shaojie Feng, Yuanxin Xing, Tianjiao Wang, Nana Huang, Ka Li, Aijun Zhang

**Affiliations:** ^1^ Department of Pediatrics, Qilu Hospital of Shandong University, Jinan, China; ^2^ Research Center of Basic Medicine, Central Hospital Affiliated to Shandong First Medical University, Jinan, China; ^3^ School of Food Science and Engineering, Qingdao Agricultural University, Qingdao, China; ^4^ Research Center of Translational Medicine, Central Hospital Affiliated to Shandong First Medical University, Jinan, China; ^5^ Department of Neurology, Central Hospital Affiliated to Shandong First Medical University, Jinan, China; ^6^ Department of Orthopedics, Qilu Hospital of Shandong University, Jinan, China

**Keywords:** osteosarcoma, bioinformatics analysis, SLC25A4, prognosis, survival analysis

## Abstract

**Background:**

Osteosarcoma (OS) is highly malignant and prone to local infiltration and distant metastasis. Due to the poor outcomes of OS patients, the study aimed to identify differentially expressed genes (DEGs) in OS and explore their role in the carcinogenesis and progression of OS.

**Methods:**

RNA sequencing was performed to identify DEGs in OS. The functions of the DEGs in OS were investigated using bioinformatics analysis, and DEG expression was verified using RT-qPCR and Western blotting. The role of *SLC25A4* was evaluated using gene set enrichment analysis (GSEA) and then investigated using functional assays in OS cells.

**Results:**

In all, 8353 DEGs were screened. GO and KEGG enrichment analyses indicated these DEGs showed strong enrichment in the calcium signaling pathway and pathways in cancer. Moreover, the Kaplan-Meier survival analysis showed ten hub genes were related to the outcomes of OS patients. Both *SLC25A4* transcript and protein expression were significantly reduced in OS, and GSEA suggested that *SLC25A4* was associated with cell cycle, apoptosis and inflammation. *SLC25A4*-overexpressing OS cells exhibited suppressed proliferation, migration, invasion and enhanced apoptosis.

**Conclusion:**

*SLC25A4* was found to be significantly downregulated in OS patients, which was associated with poor prognosis. Modulation of *SLC25A4* expression levels may be beneficial in OS treatment.

## 1 Introduction

Osteosarcoma (OS) is an aggressive malignancy of bone tissues, most common in children and adolescents. (Beird et al., 2022). The incidence of OS peaks around puberty, with an overall incidence of 3.8 per 1,000,000 people, with males slightly outnumbering females. (Zarghooni et al., 2023). OS mostly occurs in the extremities, especially in the distal femur, proximal tibia, and proximal fibula. (Kansara et al., 2014). In recent years, neoadjuvant chemotherapy for patients with limited OS has proved to be very effective; however, the prognosis for patients with distant metastases and recurrence remains dismal, with an average 5-year survival rate of about 20%. (Lillo Osuna et al., 2019; Ji et al., 2024). Although combination therapies including chemotherapy, radiotherapy and immunotherapy have been increasingly used in cancer treatment in recent years, the prognosis for patients with early metastasis and recurrent tumors is still not optimistic. (Wu et al., 2024). The survival outcomes of patients can be improved by early surveillance and diagnosis of tumors. (Villani et al., 2016). In recent years, screening for genetic changes has led to the identification of DEGs and functional pathways involved in tumor development, providing a biological basis for early diagnosis and treatment strategies. (Wang et al., 2022; Zhang et al., 2022).

The ADP/ATP transporter protein, adenine nucleotide translocator 1 (ANT1) is encoded by the solute carrier family 25 member 4 (*SLC25A4*) gene. The *SLC25A4* gene is located on the subterminal region of chromosome 4q and is highly expressed mainly in skeletal muscles, brain, and heart tissues. (Cimadamore-Werthein et al., 2023). ANT1 regulates mitochondrial energy metabolism, which facilitates the exchange of ADP and ATP between the cytoplasm and the mitochondrial matrix. (Kunji et al., 2020). Therefore, any defect in or dysfunction of the ANT1 protein can cause severe impairment of mitochondrial energy metabolism, with different degrees of impact on tissues or cells. (Kunji and Ruprecht, 2020). On the other hand, ANT1 takes part in the regulation of programmed cell death as part of the mitochondrial permeability transition pore (mPTP). (Vial et al., 2020). Dysregulation of pathways associated with programmed cell death plays a key role in cancer development and treatment resistance; therefore, reduced levels of ANT1 expression may contribute to disruptions in tumor cell apoptosis. (Brown and Attardi, 2005). A study on rhabdomyosarcoma showed that reduced ANT1 expression could affect mitochondrial function and was involved in tumor cell metabolism and death pathways, leading to tumorigenesis, which indicated that ANT1 might be a therapeutic target for rhabdomyosarcoma. (Vial et al., 2020). OS and rhabdomyosarcoma are both sarcomas; however, whether the expression levels of ANT1 influence the development of OS is yet unknown.

Herein, we identified predictors that influenced the prognosis of patients with OS and clarified their roles in OS development. The RNA-sequencing data from human OS tissues and nearby non-cancerous tissues was analyzed to identify DEGs, and we explored the expression level of *SLC25A4* in OS and the effect of changes in its expression level on OS cell proliferation, invasion, migration, and apoptosis.

## 2 Materials and methods

### 2.1 Collection of human clinical specimens

The four OS tissue specimens along with their adjacent non-cancerous tissues used for RNA sequencing were taken from the primary tumor sites of patients diagnosed with OS by Qilu Hospital of Shandong University, and their clinical chart information was shown in Table 1. All participants provided written informed consent, and the study received ethical approval from the review board of the participating institution.

**TABLE 1:** De-identified clinical data of osteosarcoma patients.

Patient	Gender	Age	Incidence site	Amputation	Metastasis
osteosarcoma patient 1	male	17	left femur	YES	YES
osteosarcoma patient 2	female	14	right fibula	YES	NO
osteosarcoma patient 3	male	10	right femur	NO	NO
osteosarcoma patient 4	male	17	left femur	NO	NO

### 2.2 RNA sequencing

TRIzol (Accurate Biology, Hunan, China) was utilized for RNA isolation and purification of the samples. Subsequently, NanoDrop ND-1000 (NanoDrop, Wilmington, DE, USA) was employed to perform quality control on the total RNA in terms of quantity and purity. The mRNA containing PolyA (polyadenylate) was specifically captured via Dynabeads Oligo (dT) beads (Thermo Fisher, CA, USA), which underwent two series of purification. To fragment the captured transcript under high-temperature conditions (94°C for 5–7 min), we used the NEBNext^®^ Magnesium RNA Fragmentation Module (NEB, cat. E6150, USA). Following this step, cDNA synthesis from the fragmented RNA was carried out using Invitrogen SuperScript™ II Reverse Transcriptase (CA, USA). The second strand cDNA synthesis involved DNA polymerase I along with RNase H and DNTP in a buffer solution; terminal repair and poly(A) were also performed. Magnetic beads were then utilized to screen and purify fragments based on their sizes. UDG enzyme digestion followed by PCR assay yielded double-stranded DNAs with a fragment size of 300bp ± 50bp. Finally, LC-Bio Technologies (Hangzhou) Co., Ltd conducted sequencing and data analysis while R package edgeR or DESeq2 analyzed significant differences among the samples.

### 2.3 Screening for DEGs

DEGs were identified by comparing gene expression levels between OS tissues and adjacent non-cancerous tissues based on the criteria of |log fold-change (FC)| >1 .00 and *p*-value <0 .05. Venn diagrams were generated to determine DEGs that overlapped among the four pairs of specimens (http://bioinformatics.psb.ugent.be/webtools/Venn/). Additionally, heatmaps, histograms, and volcano plots were used to visualize gene expression levels in OS tissues *versus* adjacent non-cancerous tissues.

### 2.4 Annotation and functional enrichment of DEGs

The functions of the DEGs were explored using Gene Ontology (GO), a bioinformatics resource that provides information on biological process (BP), cellular component (CC), and molecular function (MF). In addition, the Kyoto Encyclopedia of Genes and Genomes (KEGG) was utilized for the identification of interactions and response networks involving DEGs.

### 2.5 Protein–protein interaction (PPI) axis analysis involving DEGs

The Search Tool for the Retrieval of Interacting Genes (STRING) database was utilized to analyze PPIs among the DEGs. STRING provides valuable information on protein interactions, including confidence scores, protein domains, and 3D structures. To identify key molecules involved in the tumorigenesis and development of OS, a PPI network was generated with STRING and was visualized with Cytoscape software, a tool specifically designed for PPI network visualization. We used the MCODE plugin to identify DEGs with high node scores and strong connectivity within the network. Additionally, we employed the Cytohubba plugin to identify hub genes using various topological analysis algorithms.

### 2.6 Survival analysis of DEGs

To elucidate the prognostic performance of the identified hub genes, we conducted survival analysis using the Kaplan-Meier (KM) method. OS samples from the GEO, TCGA, and EGA databases were classified into elevated or reduced expression cohorts according to the median expression of the hub genes. Subsequently, overall survival (OS) and recurrence-free survival (RFS) curves were plotted along with hazard ratio (HR) and 95% confidence interval (CI) calculations to assess associations between hub gene expression profiles and patient outcomes.

### 2.7 RNA isolation and RT-qPCR

Total RNA was obtained from exponentially growing cells with TRIzol (Invitrogen, Carlsbad, CA, USA). The cDNA synthesis was performed with the Evo M-MLV RT Mix Kit with gDNA Clean using RT-qPCR Ver.2 from Accurate Biotechnology (Hunan) Co., Ltd (ChangSha, China). The primer sequences can be found in Table 2. Each sample underwent three replicate runs of RT-qPCR via SYBR Green Premix Pro Taq HS RT-qPCR kit [Accurate Biotechnology (Hunan) Co., Ltd (ChangSha, China)] on a LightCycler 96 instrument (Roche, Basel, Switzerland). GAPDH served as the endogenous control for normalization and the relative gene expression were computed utilizing the 2^−ΔΔCT^ formula.

**TABLE 2:** Primer sequences used for reverse transcription-quantitative PCR.

Gene	Primer sequences (5′-3′)	Product size, bp
CASQ2	F: GCC​TCT​ACT​ACC​ATG​AGC​CG	20
	R: GCA​TCC​ACC​ATC​ACA​AAG​CC	20
DES	F: AGG​ACC​GAT​TTG​CCA​GTG​AG	20
	R: CTT​GAG​GTG​CCG​GAT​TTC​CT	20
KLHL31	F: ACA​GAG​TGT​ACG​TGA​TGG​GC	20
	R: CTT​CTT​CTC​GCC​CTC​GTT​CC	20
MYBPC2	F: TGT​GTT​CAA​GTG​CGA​GGT​GT	20
	R: CAG​CTT​GTG​GAA​CCT​GCC​TA	20
SYNPO2L	F: CGG​CAT​CAG​CCC​TAT​CAA​CT	20
	R: AGT​GGA​AAA​CCG​GCG​AAT​CT	20
TNNT1	F: TCA​AGG​CAG​AAC​AGA​AGC​GT	20
	R: GCT​GTT​CCT​CCC​CCA​TGT​AG	20
CASQ1	F: CCC​TAC​ATC​CCC​TTC​TTC​GC	20
	R: GCT​CCT​CCA​CGA​AGT​TGA​CA	20
SLC25A4	F: GTT​CCT​CAC​CGC​AGC​TAC​TT	20
	R: CAA​TGA​TGG​TAT​GGC​GTG​CG	20
PDLIM3	F: GAC​AAA​TGT​GGG​AGT​GGC​ATA​GT	23
	R: TGC​AGA​GAC​TTA​AGC​TTT​GGG​AT	23
MYL3	F: ACA​CCT​GAG​CAG​ATT​GAA​GAG​TT	23
	R: GGC​AGG​AAA​GTT​TCA​AAG​TCC​AT	23
GAPDH	F: GCA​CCG​TCA​AGG​CTG​AGA​AC	20
	R: TGG​TGA​AGA​CGC​CAG​TGG​A	19

F, forward; R, reverse

### 2.8 Western blot analysis

Protein samples were prepared from collected cells and subjected to SDS gel electrophoresis before being transferred onto nitrocellulose membranes, which were then blocked at room temperature (RT) for 2 h in 5% skim milk, before overnight incubation with primary antibodies obtained from ImmunoWay Biotechnology Company (Plano, TX, USA). Subsequently, they underwent three washes with Tween 20 in Tris‐buffered saline (TBST) before a 1–2 h incubation at RT in secondary antibodies. After an additional three washes of the membranes, chemiluminescence reagents were used to visualize protein expression levels. The following antibodies were used:anti-β-actin (1:10000; Proteintech, 66009-1-Ig); anti-Vinculin (1:10000; Abways, CY5164); anti-SLC25A4 (1:1000; ABclonal, A15027); anti-CASQ1 (1:1000; ABclonal, A19640); anti-CASQ2 (1:2000; Proteintech, 18422-1-AP).

### 2.9 TCGA database

The cancer genomic atlas (TCGA) database, known for its comprehensive functionalities such as analyzing differential expression, survival rates, correlations, and identifying similar genes, was utilized to assess hub gene profiles in several tumor tissues and their matched healthy tissues based on patient data.

### 2.10 Protein expression and cellular localization

The HPA database offers valuable information regarding the distribution of proteins within different cancer types based on cellular and histopathological characteristics. This resource was employed for bioinformatics data mining purposes to present confocal images showcasing the cellular localization of hub genes.

### 2.11 Analysis of the mutation and CNVs in DEGs

To explore genetic alterations, mutations, and associated prognosis related to *SLC25A4*, we utilized the cBioPortal for Cancer Genomics (http://cbioportal.org). This web resource allows for multidimensional analysis and visualization of genomics data alongside clinical features from diverse cancer samples. For our study, we chose the whole genome pan-cancer analysis (ICGC/TCGA, Nature 2020). We obtained information about copy number variations (CNVs) in hub genes found in both cancerous tissues and cell lines.

### 2.12 Gene set enrichment analysis (GSEA)

Transcriptional data from GSE238110 was downloaded and the subjects were divided into elevated and reduced expression cohorts according to the median *SLC25A4* contents, and then the inter-cohort differences were assessed by limma. Next, we employed GSEA to identify the DEG-associated signaling networks that are significant between the elevated- and reduced-*SLC25A4* expression cohorts. (Subramanian et al., 2005).

### 2.13 Cell culture and transfection

The human OS cell lines MG-63, HOS, and Saos-2 were purchased from Genechem Co., Ltd (Shanghai, China), Procell Life Science & Technology Co., Ltd. (Wuhan, China), and BOSTER Biological Technology Co., Ltd (Wuhan, China), respectively. The human osteoblast cell line hFOB1.19 was obtained from the Institute of Biochemistry and Cell Biology, Chinese Academy of Sciences (Shanghai, China). MEM (Procell, Wuhan, China) was used for HOS and MG-63 cell culture, and McCoy’s 5A medium (BOSTER, Wuhan, China) was used for the culture of Saos-2 cells. Moreover, DMEM/F12 medium (Gibco, MA, USA) was employed for the hFOB1.19 cell culture. All media contained 10% fetal bovine serum (FBS, Gibco) and were grown in an atmosphere containing 5% CO_2_. OS cells were maintained at 37°C, while the hFOB1.19 cells were cultured at 34°C. Transient transfection of siRNA (RiboBio, Guangzhou, China), plasmids and the control was conducted with Lipofectamine 2000 (Invitrogen) following the provided directions. Both RT–qPCR and Western blotting were used to verify the transfection efficiency.

### 2.14 Cell counting kit (CCK)-8 assays

CCK-8 assays (Yeasen Biotechnology, Shanghai, China) were used to assess the proliferation of OS cells. HOS and MG-63 cells (1 × 10^5^ per well) were seeded in 96-well plates and incubated for 24, 48, 72, 96, or 120 h. CCK-8 solution (10 μL per well) was then added and incubated at 37°C for 1 h. Absorbances at 450 nm were read in an Epoch microplate spectrophotometer (SpectraMax i3x).

### 2.15 EdU assays

Cell proliferation rates were assessed by the Cell Light™ EdU Apollo567 *In Vitro* Kit (RiboBio). Briefly, cells cultured in 96-well plates were transfected for 48 h. Next, EdU (50 μM) was added to the cells and incubated (2 h, 37°C). The cells were then fixed in 4% formaldehyde for 0.5 h, followed by incubation with glycine (2 mg/mL) for 5 min and a 10-min cell permeabilization with 0.5% Triton X-100, before washing in PBS, incubation with the Apollo reaction cocktail for 30 min incubation, and washed twice with 0.5% Triton X-100. The cells were then stained with Hoechst 33342 for 30 min and evaluated under fluorescence microscopy (Olympus, Japan).

### 2.16 Wound healing assessment

After transfection, cells were plated into 6-well plates and grown to 100% confluency. Wounds were operated with 200 μL tips in the middle of the wells, rinsed three times using PBS, and then the medium was changed with serum-free medium immediately. Image capture utilized an inverted phase-contrast microscope (OLYMPUS, Japan) at 0 h and 24 h, respectively.

### 2.17 Transwell evaluation

Transwell chambers (8 µm pore size; Corning, NY, USA) were used to examine migration and invasion in OS cells. Cells (1 × 10) were placed in the top chamber which was precoated with 50 µL 1:8 diluted Matrigel (Corning) (to test invasion) or not (to test migration). Cells in the top chamber were in 200 µL of serum-free culture medium whereas cells in the bottom chamber were cultured in medium with 10% FBS. Following 37 °C incubation for 24 h, the cells in the lower chamber were fixed with 4% paraformaldehyde followed by staining with crystal violet for 1 h at RT. Cell numbers in five randomly selected fields were recorded. All the above experiments were repeated three times.

### 2.18 Flow cytometry

Apoptosis was assessed in treated OS cells after staining with Annexin V-FITC/PI. After cell precipitation and resuspension, the cells were exposed to 5 μL Annexin V-FITC and 10 μL PI Staining solution, and incubated in the dark at RT for 10–15 min. Then, samples were resuspended in 400 μL 1×Binding Buffer, placed on ice, and evaluated by flow cytometry (BD LSRFortessa) within 1 h.

### 2.19 Statistical analysis

All data were statistically analyzed using GraphPad Prism eight software. Analysis of the differences between the two groups was determined by Student’s t-test. A *p*-value of <0.05 was considered statistically significant.

## 3 Results

### 3.1 RNA sequencing analysis of tumor tissues and adjacent non-cancerous tissues

**FIGURE 1:**
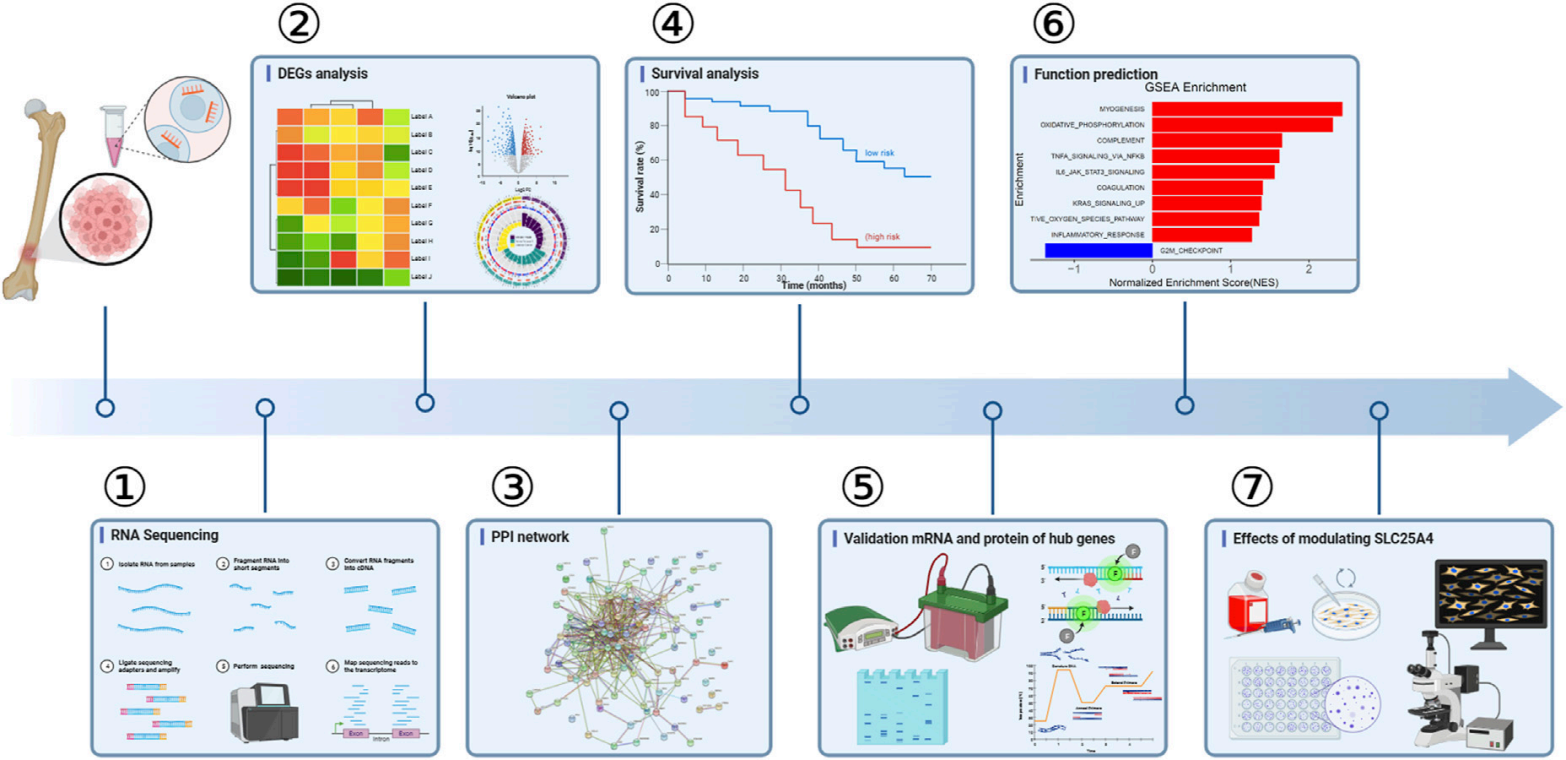
Design of the study. DEGs, differentially expressed genes; PPI, protein-protein interaction.

**FIGURE 2:**
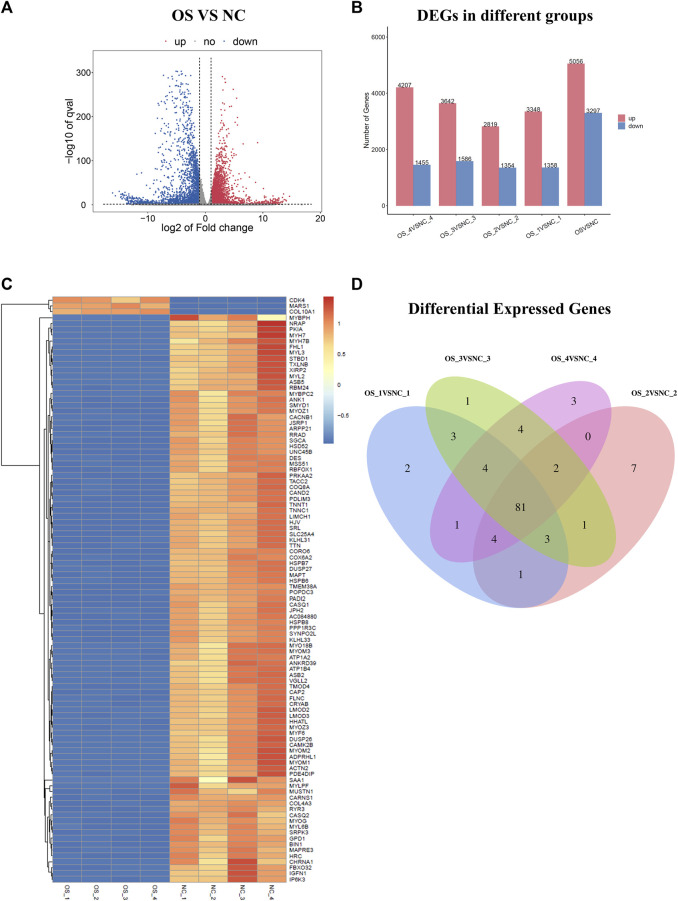
Identification of DEGs in osteosarcoma. **(A)** Volcano plots of DEG distribution. **(B)** DEG distribution in four pairs of tissues. **(C)** Heatmap of the top 100 DEGs. **(D)** Venn diagram showing the intersection of four pairs of tissues. Red and blue colors represent upregulated and downregulated DEGs, respectively. DEGs, differentially expressed genes.

In this study, we combined RNA sequencing, bioinformatics, and experimental results for multilevel verification of the identification of key genes in OS. The study design is shown in Figure 1. The RNA-sequencing results of OS and adjacent non-cancerous tissues were analyzed and DEGs were identified using the criteria of |Log2 FC| >1 and *p*-value <0.05 (Figure 2A). A total of 8353 DEGs were identified between OS and normal tissues, of which 5,056 were upregulated and 3,297 were downregulated (Supplementary Figure S1). There were 4,706 DEGs in OS 1, including 3,348 upregulated and 1358 downregulated genes. In all, 4,173 DEGs were identified in OS 2, of which 2,819 were upregulated and 1354 were downregulated. Additionally, 5,228 DEGs were identified in OS 3, including 3,642 upregulated and 1586 downregulated genes, while 5,662 DEGs were found in OS 4, with 4,207 upregulated and 1455 downregulated (Figure 2B). The top 100 DEGs were visualized using a heatmap, with red color indicating upregulation and blue downregulation (Figure 2C). The Venn diagrams showed that among the top 100 DEGs, there were 81 genes that were common to the four pairs of specimens (Figure 2D).

### 3.2 KEGG and GO enrichment analyses involving DEGs

**FIGURE 3:**
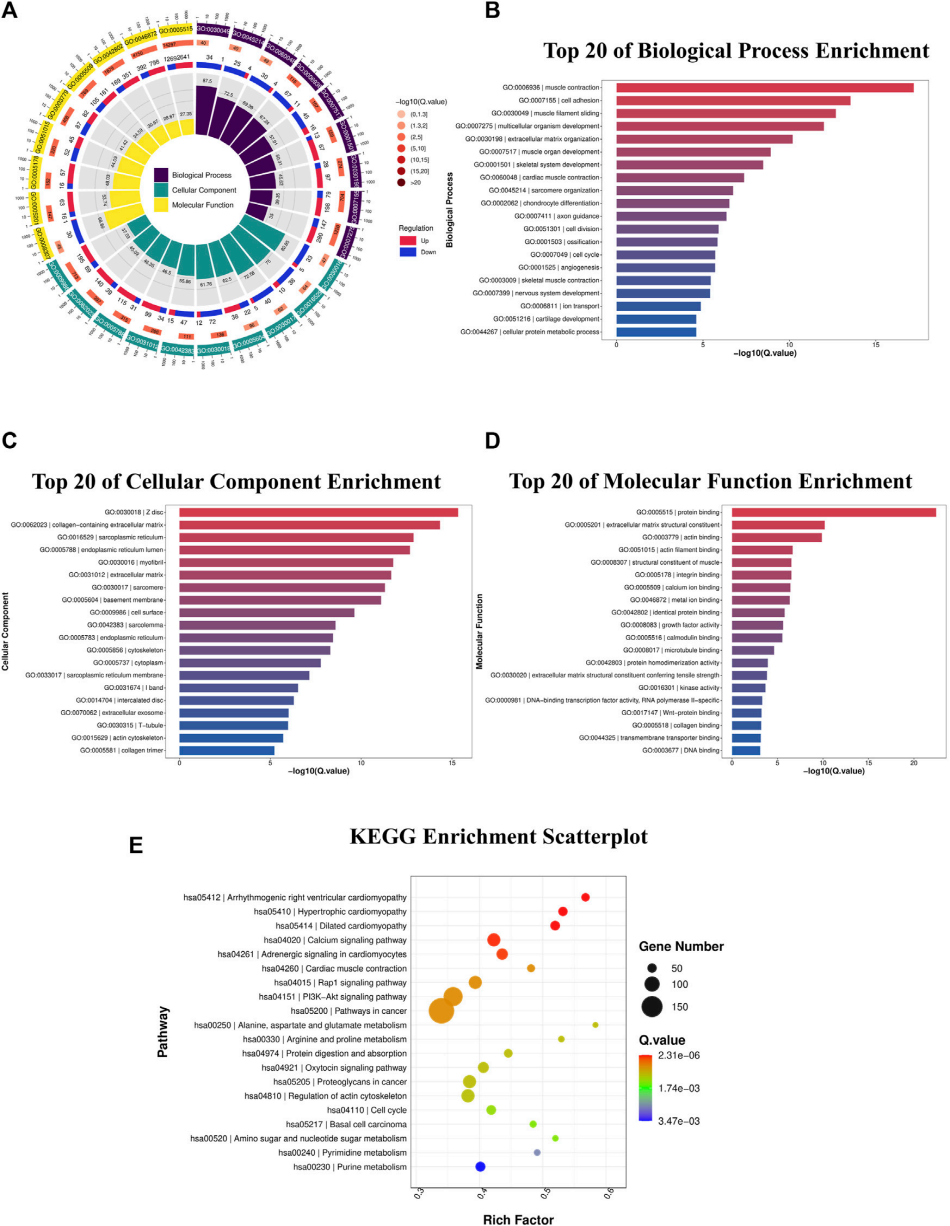
DEG functional enrichment analysis. **(A)** DEG GO enrichment assessment in the BP, CC, and MF categories. **(B)** Top 20 enriched BPs. **(C)** Top 20 enriched CCs. **(D)** Top 20 enriched MFs. **(E)** DEG KEGG pathway enrichment assessment. DEGs, differentially expressed genes; GO, Gene Ontology; BP, biological process; CC, cellular component; MF, molecular function; KEGG, Kyoto Encyclopedia of Genes and Genomes.

The biological roles of DEGs in OS tumorigenesis and metastasis were explored using KEGG and GO enrichment analyses. The BP, CC, and MF were analyzed using GO enrichment analyses (Figure 3A; Table 3). In BP analysis, DEGs showed predominant enrichment in muscle contraction, cell adhesion, and muscle filament sliding (Figure 3B). The CC analysis indicated that the DEGs played a role in Z disc, collagen-containing extracellular matrix, and sarcoplasmic reticulum (Figure 3C). Moreover, the MF analysis revealed that the DEGs showed major contribution in protein interaction, extracellular matrix structural component, and actin-binding (Figure 3D). KEGG network enrichment assessment revealed that most DEGs contributed to the calcium, cancer-related, Rap1 and PI3K-Akt networks (Figure 3E; Table 4). The above results indicated that these DEGs were potential regulators of OS initiation and progression.

**TABLE 3:** Top 15 enriched GO terms of DEGs.

Category	Term	Count	Adjusted *p*-value
BP	muscle contraction	78	6.35093887884101E-18
BP	cell adhesion	277	2.89817618033311E-14
BP	muscle filament sliding	35	2.05144935275006E-13
BP	multicellular organism development	427	1.00611226412926E-12
BP	extracellular matrix organization	125	6.46066513499453E-11
CC	Z disc	84	4.58043759857695E-16
CC	collagen-containing extracellular matrix	179	4.57418130860211E-15
CC	sarcoplasmic reticulum	48	1.3010896884917E-13
CC	endoplasmic reticulum lumen	146	2.05144935275006E-13
CC	myofibril	38	0000000000017214363
MF	protein binding	3,910	3.64993423987298E-23
MF	extracellular matrix structural constituent	79	6.46066513499453E-11
MF	actin binding	169	1.33271772180396E-10
MF	actin filament binding	97	2.16177351583286E-07
MF	integrin binding	73	2.95877318627355E-07

BP, biological process; CC, Cellular component; GO, Gene Ontology; MF, molecular function

**TABLE 4:** Top 15 enriched KEGG pathway terms of DEGs.

Pathway	ID	Gene count	Adjusted *p*-value
Hypertrophic cardiomyopathy	hsa05410	50	2.31333E-06
Dilated cardiomyopathy	hsa05414	53	2.31333E-06
Arrhythmogenic right ventricular cardiomyopathy	hsa05412	42	2.31333E-06
Calcium signaling pathway	hsa04020	82	4.8789E-05
Adrenergic signaling in cardiomyocytes	hsa04261	68	9.46452E-05
Pathways in cancer	hsa05200	189	0.0005978319
Rap1 signaling pathway	hsa04015	83	0.000578319
PI3K-Akt signaling pathway	hsa04151	134	0.000578319
Cardiac muscle contraction	hsa04260	39	0.000578319
Oxytocin signaling pathway	hsa04921	65	0.001017813
Arginine and proline metabolism	hsa00330	27	0.001017813
Proteoglycans in cancer	hsa05205	83	0.001017813
Alanine, aspartate and glutamate metabolism	hsa00250	21	0.001017813
Protein digestion and absorption	hsa04974	45	0.001017813
Regulation of actin cytoskeleton	hsa04810	84	0.001055331

### 3.3 Screening hub genes using protein–protein interaction (PPI) and module assessment

**FIGURE 4:**
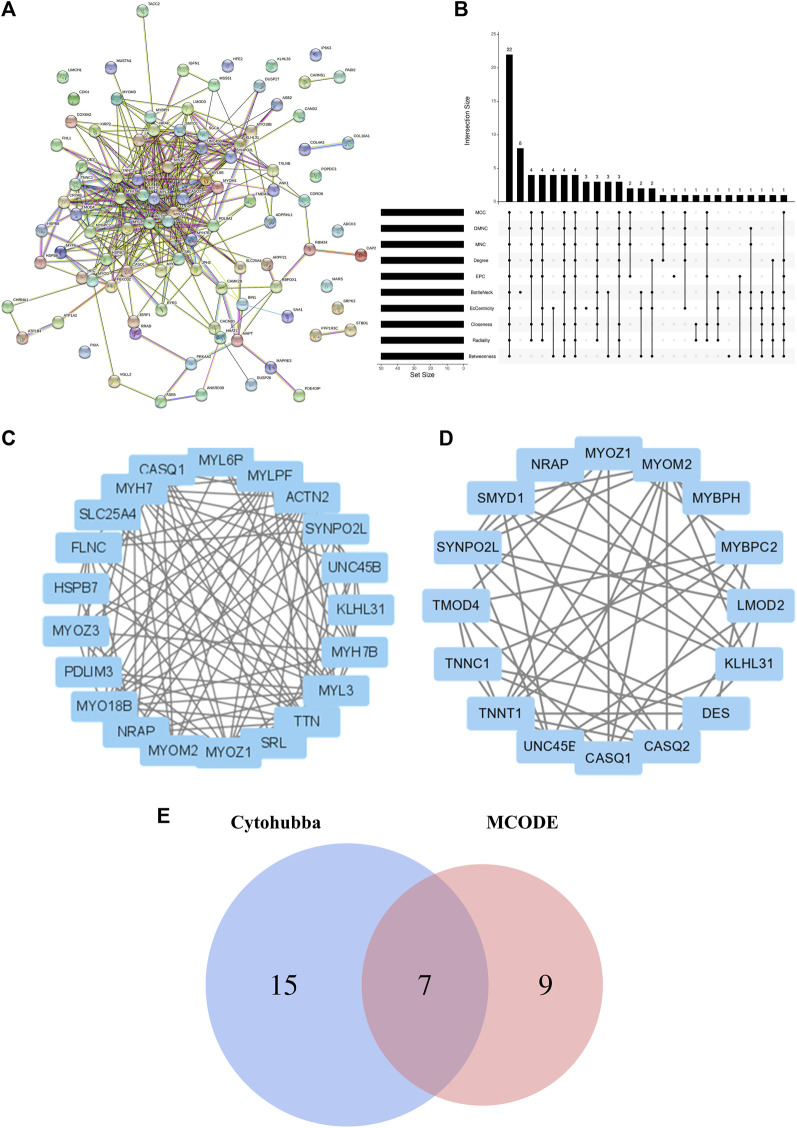
Construction of the PPI network, evaluation of important modules, and identification of hub genes. **(A)** Whole PPI betwork. **(B)** Hub genes identified via intersection of 50 genes from 10 algorithms using Cytohubba, namely, MCC, DMNC, MNC, Degree, EPC, BottleNeck, EcCentricity, Closeness, Radiality, and Betweenness. **(C)** Hub genes identified by Cytohubba. **(D)** Module 1 PPI netowrk. **(E)** Venn diagram showing the intersection of 22 common genes using Cytohubba and genes within the most significant module. PPI, protein-protein interaction.

The interactions among the DEGs were further explored via the STRING database. A PPI of three upregulated and 94 downregulated genes was constructed; this consisted of 97 nodes and 377 edges (Figure 4A). Various algorithms for topological analysis, including MCC, DMNC, MNC, degree, EPC, bottleNeck, EcCentricity, closeness, radiality, and betweenness, were used to predict and identify key nodes in the PPI network using Cytohubba. This led to the identification of 50 top genes (Figure 4B). The interactions between these genes were then evaluated, identifying 22 hub genes, including *SLC25A4*, *ACTN2*, *TTN*, *MYL3*, *MYH7*, *MYOM2*, *MYOZ1*, *UNC45B*, *SYNPO2L*, *KLHL31*, *MYLPF*, *NRAP*, *CASQ1*, *PDLIM3*, *MYL6B*, *FLNC*, *MYH7B*, *MYO18B*, *SRL*, *TXLNB*, *MYOZ3*, and *HSPB7* (Figure 4C; Supplementary Figure S2). Hub genes were recognized via the MCODE plugin in Cytoscape, with the most important module containing 16 hub genes (Figure 4D). Among them, seven genes coincided with the hub genes in the Cytohubba plugin (Figure 4E).

### 3.4 Hub genes-associated overall survival assessment

**FIGURE 5:**
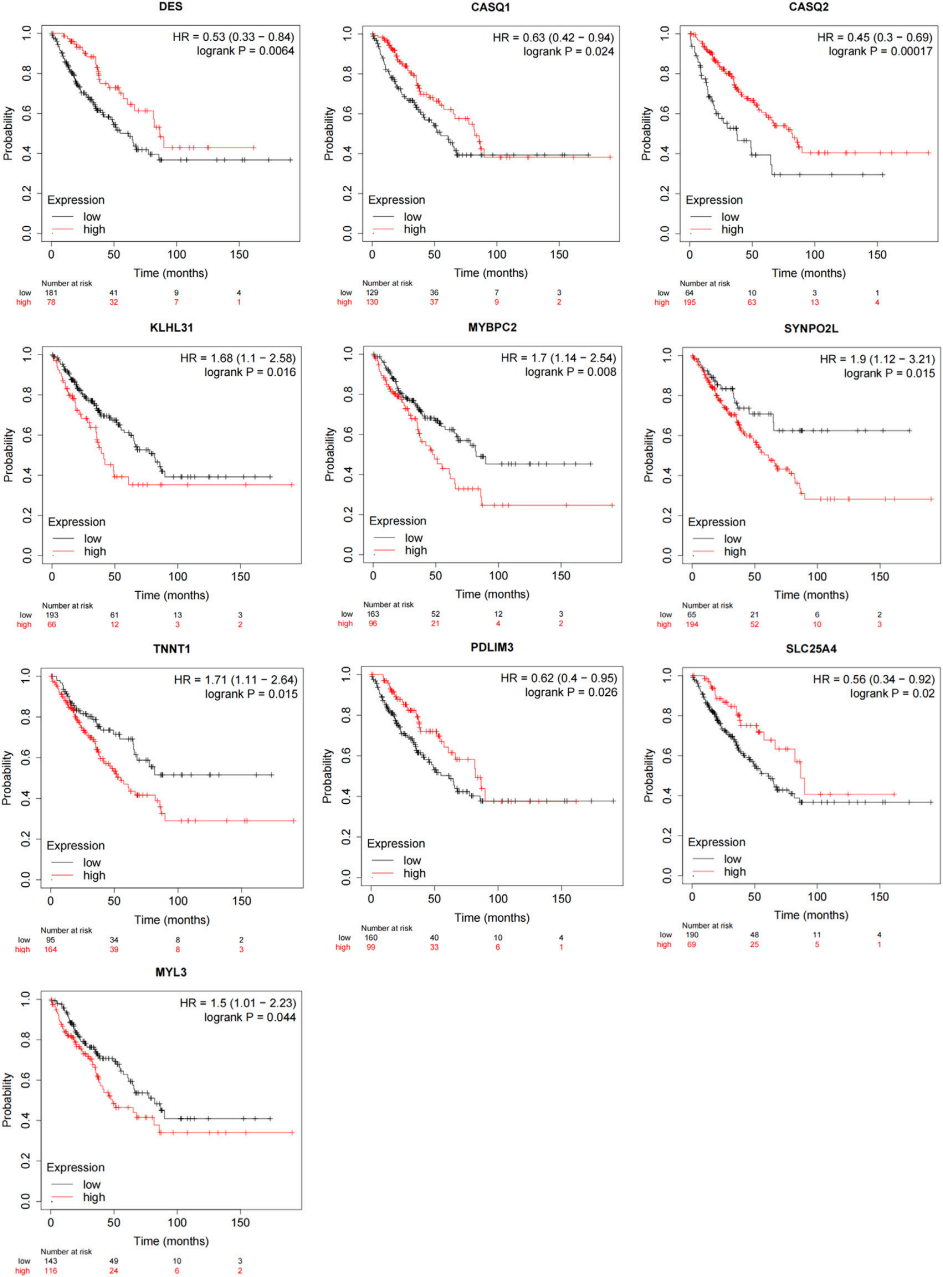
Prognostic assessment of hub genes via the Kaplan-Meier plotter. Changes in *DES*, *CASQ1*, *CASQ2*, *KLHL31*, *MYBPC2*, *SYNPO2L*, *TNNT1*, *PDLIM3*, *SLC25A4*, and *MYL3* genes were intimately linked to OS patient overall survival (*p* < 0.05).

K-M survival curves were used to confirm the associations between the hub genes and OS patient survival outcomes (Figure 5; Supplementary Figure S3). The findings showed that increased expression of *CASQ1* (HR = 0.63 [0.42–0.94], *p* = 0.024), *CASQ2* (HR = 0.45 [0.3–0.69], *p* = 0.00017), *DES* (HR = 0.53 [0.33–0.84], *p* = 0.0064), *PDLIM3* (HR = 0.62 [0.4–0.95], *p* = 0.026), and *SLC25A4* (HR = 0.56 [0.34–0.92], *p* = 0.02) had better overall survival, while those with higher expression of *KLHL31* (HR = 1.68 [1.1–2.58], *p* = 0.016), *MYBPC2* (HR = 1.7 [1.14–2.54], *p* = 0.008), *SYNPO2L* (HR = 1.9 [1.12–3.21], *p* = 0.015), *TNNT1* (HR = 1.71 [1.11–2.64], *p* = 0.015), and *MYL3* (HR = 1.5 [1.01–2.23], *p* = 0.044) had worse overall survival (Figure 5). The abbreviations, full names, and functions of these 10 genes are provided in Supplementary Table S1. In conclusion, these results suggested that the expression of these genes can be indicative of prognosis in OS patients and might act as significant prognostic biomarkers for OS patients.

### 3.5 Validation of hub gene mRNA and protein contents in OS

**FIGURE 6:**
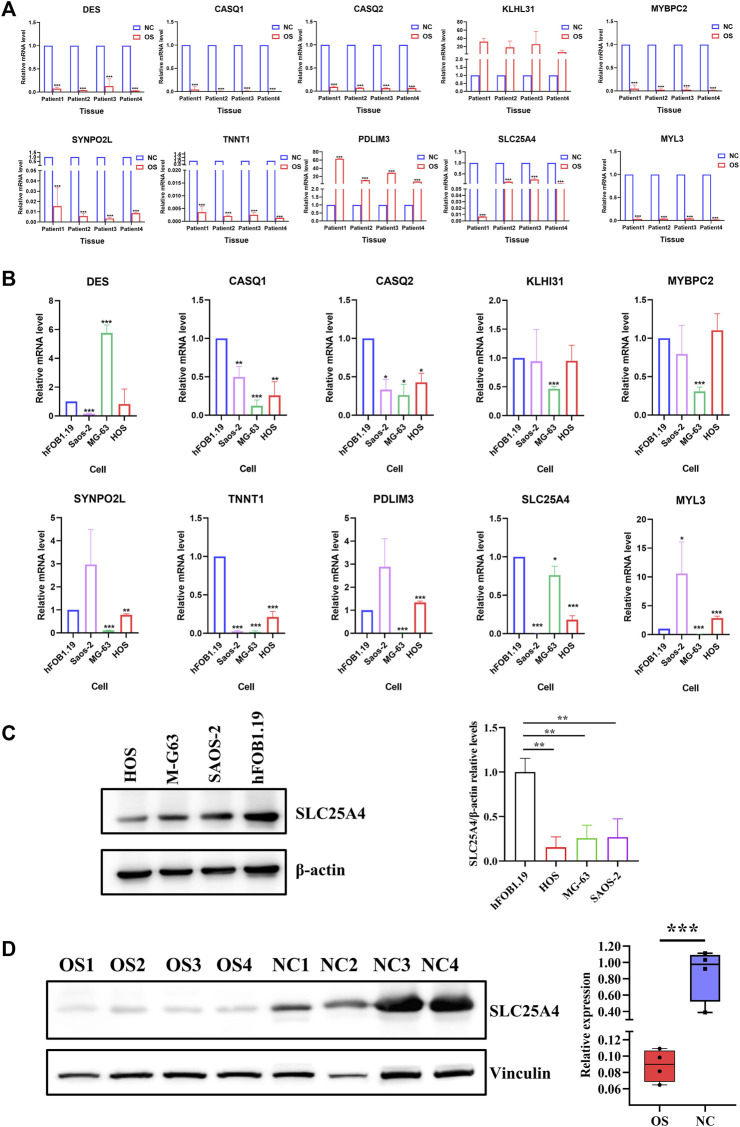
Hub gene transcript and protein expressions in OS. **(A)** Transcript levels of *DES*, *CASQ1*, *CASQ2*, *KLHL31*, *MYBPC2*, *SYNPO2L*, *TNNT1*, *PDLIM3*, *SLC25A4*, and *MYL3* in OS tissues relative to surrounding non-cancerous tissues were verified using real-time qPCR. **(B)** mRNA expression levels of *DES*, *CASQ1*, *CASQ2*, *KLHL31*, *MYBPC2*, *SYNPO2L*, *TNNT1*, *PDLIM3*, *SLC25A4*, and *MYL3* in human OS cell lines (MG-63, HOS, and Saos-2) compared to human fetal osteoblast cell line (hFOB1.19) were verified using real-time qPCR. **(C)** Basal expression of SLC25A4 in OS cells and normal cell line HFOB examined by Western blot. **(D)** Results of Western blot on SLC25A4 expression in OS tissues compared to surrounding healthy tissues. n ≥ 3, *p < 0.05, **p < 0.01, ***p < 0.001.

Table 5 showed the transcript profiles of the ten genes obtained by RNA sequencing. Differences among hub gene transcript expressions in OS were further explored by RT-qPCR. The results showed that *DES, CASQ1, CASQ2, MYBPC2, SYNPO2L, TNNT1, SLC25A4*, and *MYL3* had reduced expression in OS tissues, which consistent with our RNA sequencing results. Whereas *PDLIM3* and *KLHL31* were significantly upregulated at the expression levels in OS tissues (*p* < 0.05; Figure 6A). Moreover, we also validated the ten gene expression profiles in OS cell lines. Expression levels of *CASQ1, CASQ2, SLC25A4,* and *TNNT1* were significantly lower in all human OS cells, including MG-63, HOS, and Saos-2, relative to the human fetal osteoblast cell line (hFOB1.19) (Figure 6B). Furthermore, their contents in OS and normal tissues were also demonstrated with TCGA. The expression levels of *CASQ1*, *CASQ2*, and *SLC25A4* were downregulated in OS, and these results corroborated with the RT-qPCR data (Supplementary Figure S4A). In addition to the expression levels of mRNA, the hub gene protein expressions in hFOB1.19, MG-63, HOS, and Saos-2 cells were further verified using Western blot. We revealed that the *SLC25A4* protein expression in MG-63, HOS, and Saos-2 were lower relative to hFOB1.19, while the *CASQ1* and *CASQ2* protein contents did not decrease significantly (Figure 6C; Supplementary Figure S4B). Then, we validated the expression levels of SLC25A4 in four paired OS samples with adjacent normal tissues. Based on these findings, SLC25A4 was strongly diminished among the tumor group (Figure 6D).

**TABLE 5:** Differential expression of hub genes between osteosarcoma tissues and adjacent non-cancerous tissues.

Gene	Gene ID	Adjusted *p*-value	Log (FC)	Regulation
DES	ENSG00000175084	0	−6.24	Hypo
CASQ1	ENSG00000143318	0	−8.05	Hypo
CASQ2	ENSG00000118729	0	−5.11	Hypo
KLHL31	ENSG00000124743	0	−5.73	Hypo
MYBPC2	ENSG00000086967	0	−5.79	Hypo
SYNPO2L	ENSG00000166317	0	−7.28	Hypo
TNNT1	ENSG00000105048	0	−8.89	Hypo
PDLIM3	ENSG00000154553	0	−5.25	Hypo
SLC25A4	ENSG00000151729	0	−4.21	Hypo
MYL3	ENSG00000160808	0	−5.76	Hypo

### 3.6 Localization and function of SLC25A4 in OS

**FIGURE 7:**
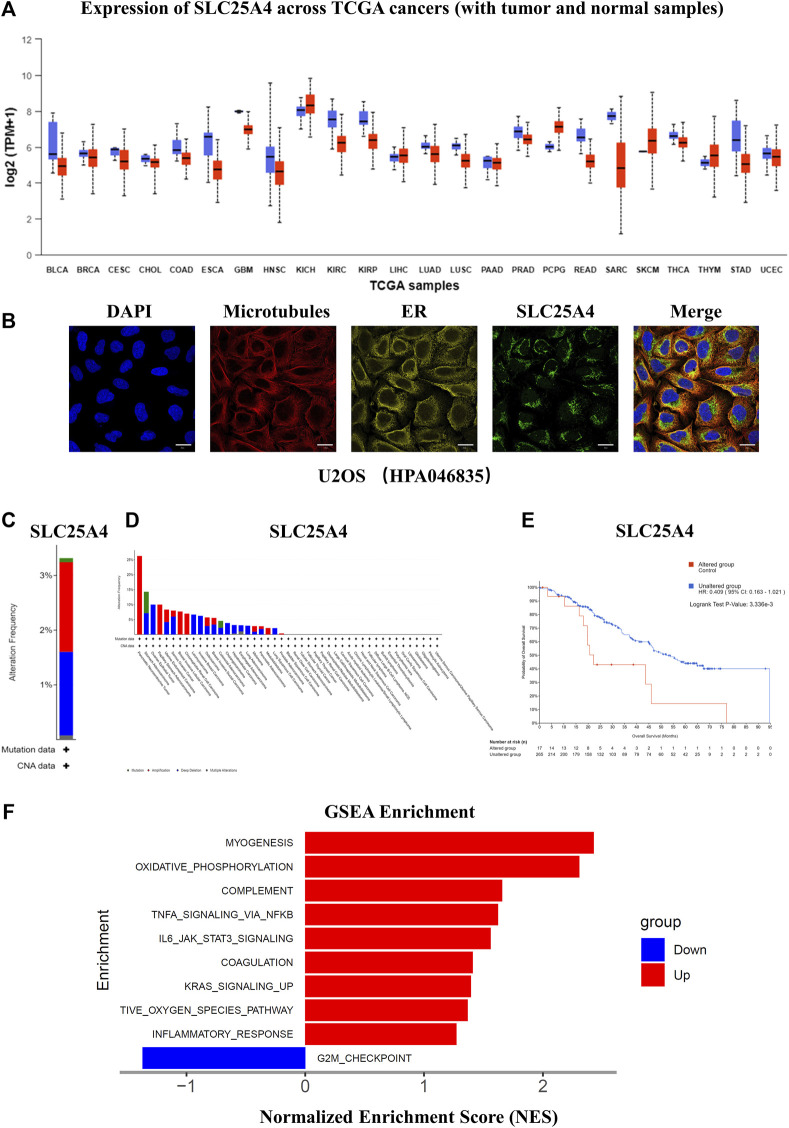
Expression of *SLC25A4* in different cancers and function of the *SLC25A4* in the OS. **(A)** Expression of *SLC25A4* across TCGA cancers. **(B)** The confocal images of the cellular localization of *SLC25A4* in U2OS cells (scale bar: 20 µm). **(C)** Genetic alterations and mutations of *SLC25A4* in pan-cancer. **(D)** Genetic alterations and mutations of *SLC25A4* in OS. **(E)** Genetic alterations and mutations in *SLC25A4* were strongly associated with overall survival (*p* < 0.05). **(F)** Functional and Pathway Enrichment Analysis of *SLC25A4* by GSEA.

*SLC25A4* expression in pan-cancer was then explored, which indicated a decrease in the *SLC25A4* expression in most tumors (Figure 7A). As the functions of proteins are often determined by their subcellular localization, the subcellular localization of *SLC25A4* in OS was explored using the HPA database. The confocal images revealed that *SLC25A4* was present in the mitochondria (Figure 7B). Whole genome pan-cancer analysis (ICGC/TCGA, Nature 2020) was performed to identify the genetic alterations and mutations in *SLC25A4* via the cBioPortal database. The frequencies of *SLC25A4* mutations in pan-cancer were 3%, and they were typically missense mutation, splice mutation, truncating mutation, amplification, and deep deletion (Figure 7C). Moreover, the frequencies of *SLC25A4* mutations were significantly higher in OS patients, accounting for 5.71% (Figure 7D). The data also suggested that the overall *SLC25A4*-mutated patient survival was worse relative to those with no mutations, indicating that the genetic alterations in *SLC25A4* genes was significantly associated with the prognosis of OS patients (Figure 7E). To further elucidate *SLC25A4* significance in OS, we employed GSEA to screen the leading 10 networks that showed enrichment in the elevated- and reduced-*SLC25A4* expression cohort (Figure 7F). GSEA showed that the high expression of *SLC25A4* may potentially mediate certain inflammation, apoptosis and immune-related pathways, such as Oxidative Phosphorylation, TNFα Signaling Via NFκB, IL6/JAK2/STAT3 Signaling. On the other hand, the low expression of *SLC25A4* was enriched in the G2M checkpoint, suggesting a possible association with cell proliferation. Therefore, GSEA revealed that *SLC25A4* was associated with cell cycle, apoptosis, immune response, cell proliferation and inflammation. In order to further explore the functionality of this molecule, we conducted molecular basic experiments.

### 3.7 Effects of SLC25A4 knockdown on OS cell proliferation, migration, invasion and apoptosis

**FIGURE 8:**
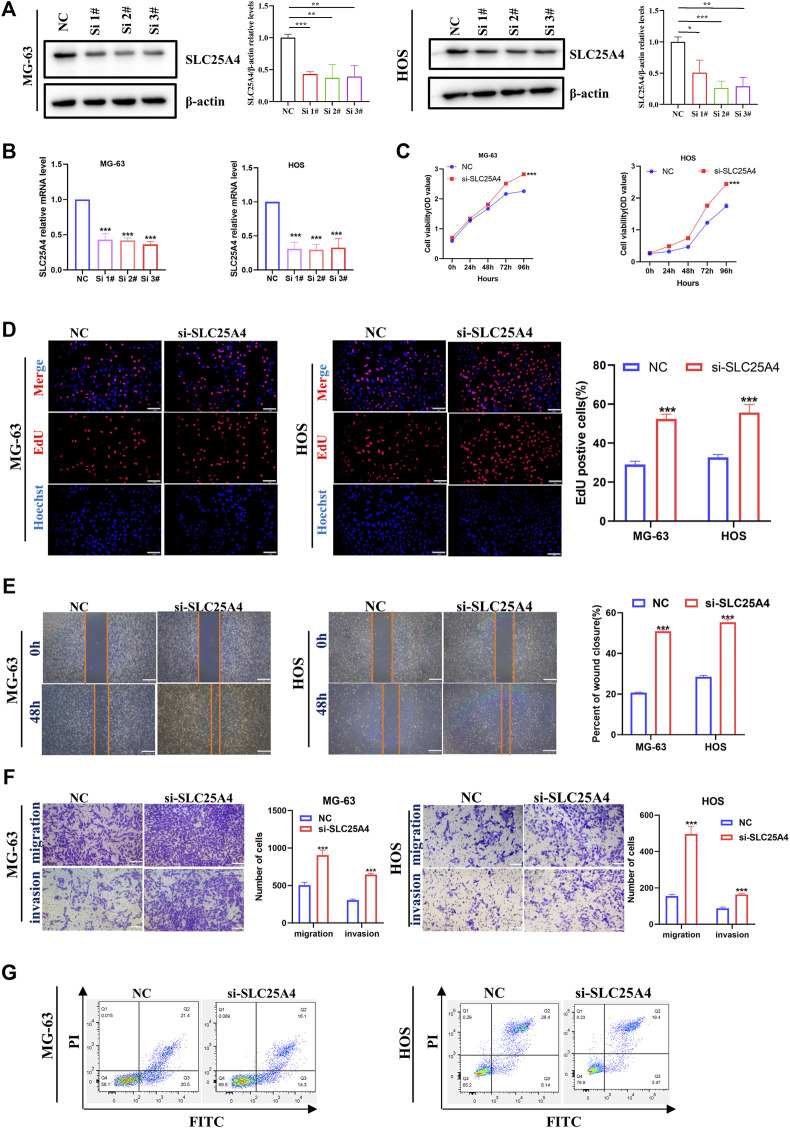
Influences of *SLC25A4* deficiency on OS cell proliferation, migration, invasion and apoptosis. Verification of transfection efficiency of siRNAs by Western blot **(A)** and RT-qPCR **(B)**. CCK-8 **(C)** and EdU **(D)** revealed that *SLC25A4* knockdown significantly accelerated OS cell proliferation (scale bar: 100 µm). **(E)** Wound healing assessment revealed that the *SLC25A4-*deficient migratory cells were considerably more than siNC groups (scale bar: 500 µm). **(F)** Transwell assessment revealed that *SLC25A4* deficiency strongly accelerated OS cell migration and invasion (scale bar: 200 µm). **(G)** *SLC25A4* knockdown inhibited apoptosis rate of HOS. n ≥ 3, *p < 0.05, **p < 0.01, ***p < 0.001.

In order to investigate the effects of *SLC25A4* knockdown on OS, the HOS and MG-63 cells were selected to establish the *SLC25A4* knockdown cells. Small interfering (si) RNAs, targeting *SLC25A4* and negative control siRNA (siNC) synthesized by RiboBio were transfected into HOS and MG-63 cells. RT-qPCR and Western blot assessments were performed to verify the effects of *SLC25A4* knockdown. Based on our results, siSLC25A4-3 exhibited the optimal knockdown effects (Figures 8A,B). Therefore, siSLC25A4-3 was selected for subsequent experiments to validate the effects of *SLC25A4* knockdown on OS.

First, we explored the *SLC25A4* knockdown-mediated effect on OS cell proliferation. CCK-8 assay was used to evaluate OS cell proliferation, which showed that the *SLC25A4* knockdown cell growth rate, including both HOS and MG-63 cells, was significantly increased (Figure 8C). Furthermore, consistent with the CCK-8 data, EdU assessment showed that the downregulation of *SLC25A4* expression significantly increased the proliferation rates of HOS and MG-63 cells (Figure 8D). Together, these findings indicated that *SLC25A4* deficiency could enhance the proliferative ability of HOS and MG-63 cells.

Given the importance of migratory and invasive features of tumor cells in tumor development and metastasis, wound healing and Transwell assessments were next conducted on *SLC25A4* knockdown cells. The wound healing assay revealed that *SLC25A4* knockdown enhanced the HOS and MG-63 cell migration (Figure 8E). Furthermore, the Transwell assay, which can be used to explore both cell migration and cell invasion, revealed that *SLC25A4* knockdown predominantly promoted the migration and invasion abilities of HOS and MG-63 cells, which were related to the metastasis of OS (Figure 8F).

ANT1, as a component of mPTP, was closely associated with apoptosis; therefore, its effect on OS cell apoptosis was assessed via flow cytometry. Knockdown of *SLC25A4* decreased the overall apoptosis rates of HOS and MG-63 cells (Figure 8G).

### 3.8 SLC25A4 overexpression-mediated regulation of OS cell proliferation, migration, invasion and apoptosis

**FIGURE 9:**
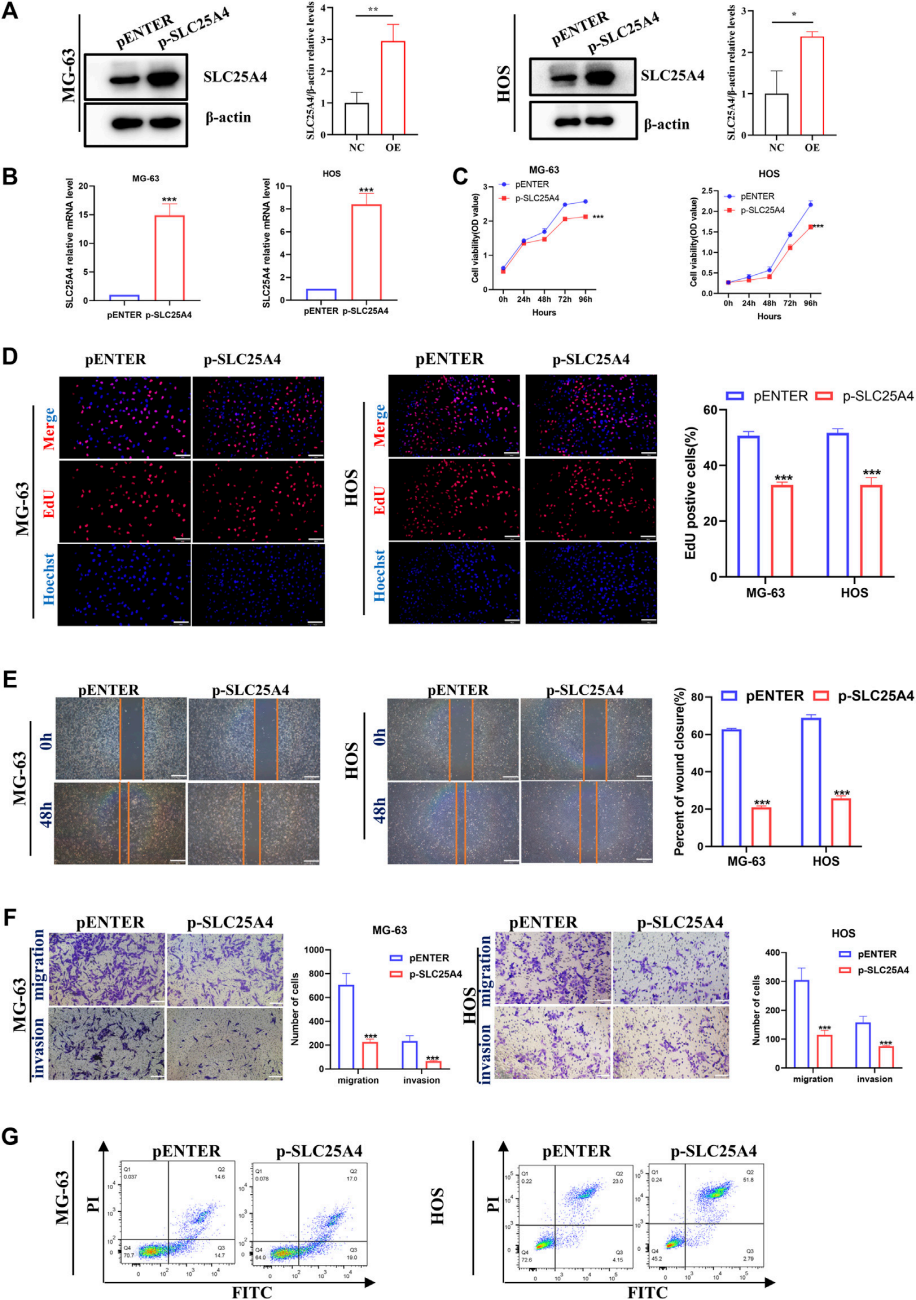
Influences of *SLC25A4* overexpression on OS cell proliferation, migration, invasion and apoptosis. Verification of transfection efficiency of plasmid by Western blot **(A)** and RT-qPCR **(B)**. CCK-8 **(C)** and EdU **(D)** revealed that *SLC25A4* overexpression significantly inhibited proliferation of OS cells (scale bar: 100 µm). **(E)** Wound healing assessment revealed that the SLC25A4-overexpressed migratory cells were considerably less compared to siNC groups (scale bar: 500 µm). **(F)** Transwell assessment revealed that *SLC25A4* overexpression significantly suppressed OS cell migration and invasion (scale bar: 200 µm). **(G)** *SLC25A4* overexpression promoted apoptosis rate of HOS. n ≥ 3, *p < 0.05, **p < 0.01, ***p < 0.001.

Since *SLC25A4* knockdown played an inhibitory role in tumorigenesis and progression of OS, we further investigated whether *SLC25A4* overexpression might have the opposite effect. The *SLC25A4*-overexpressing plasmid was used to upregulate *SLC25A4* expression in HOS and MG-63 cells. *SLC25A4* overexpression efficiency was verified using RT-qPCR and Western blot (Figures 9A,B).

*SLC25A4* expression-mediated regulation of OS cell proliferation, migration and invasion was investigated by constructing *SLC25A4*-overexpressing HOS and MG-63 cells. After the overexpression of *SLC25A4*, HOS and MG-63 cells showed a dominant inhibition of proliferation ability, which was confirmed using CCK-8 and EdU assays (Figures 9C,D). From the perspective of cell migration, wound healing assay confirmed that elevated *SLC25A4* expression levels impaired the migration ability of OS cells (Figure 9E). Additionally, the Transwell assay showed that *SLC25A4* overexpression strongly suppressed OS cell migratory and invasive abilities (Figure 9F); moreover, flow cytometry revealed that the overexpression of *SLC25A4* significantly accelerated HOS and MG-63 cell apoptosis, confirming the promise of *SLC25A4* as a potential therapeutic target for OS (Figure 9G).

## 4 Discussion

OS is a highly lethal tumor with a high incidence in children and adolescents. (Heng et al., 2024). Despite advances in the treatment of OS, including surgery and chemotherapy, overall patient survival has not improved significantly due to the unclear molecular mechanisms associated with OS tumorigenesis. Therefore, OS contributes significantly to cancer-associated mortality in children and adolescents. (Lin et al., 2017). As established biomarkers play a major role in the diagnosis and prognosis of patients, multiple investigations have been conducted to explore the potential therapeutic targets to improve prognosis. (Colli et al., 2021; Bai et al., 2023; Newhook et al., 2023). For instance, the whole-genome and RNA-sequencing data of 316 patients, obtained from the TCGA database, were employed to analyze the genomic and transcriptomic status of DNA damage response (DDR) gens in oral squamous cell carcinoma (OSCC), leading to the identification of eight hub genes, which could predict the treatment response of OSCC to novel anti-tumor compounds, thereby improving patient outcomes. (Pomella et al., 2023). Tian *et al.* identified DEGs between mice with subarachnoid hemorrhage (SAH) and control mice using two datasets, GSE167110 and GSE79416, from the GEO database and also explored the expression levels and functional pathways of the hub gene *CCR2*. (Tian et al., 2022). Hou *et al.* explored the CD74/STAT1 signaling pathway in trastuzumab-induced cardiotoxicity using GO and KEGG analysis. (Hou et al., 2023). It has been found that FBXO9 affects the metastasis of lung cancer cells by participating in the assembly process of the Vacuolar-type H + -ATPase (V-ATPase), thus influencing the prognosis of lung cancer patients. (Liu et al., 2024). Therefore, it is critical to utilize genomic-level research in the clinical practice of cancer treatment.

In this study, using comprehensive bioinformatics analysis of RNA sequencing data from OS tissues and adjacent non-cancerous tissues, we demonstrated that the mRNA and protein expression of *SLC25A4* was markedly downregulated in OS patients. Furthermore, pan-cancer analysis revealed that *SLC25A4* expression was reduced in most tumors, and the cBioPortal database indicated that genetic alterations in *SLC25A4* were strongly linked to OS patient prognosis. GSEA revealed that *SLC25A4* was associated with the cell cycle, apoptosis, the immune response, cell proliferation, and inflammation. In addition, HOS and MG-63 cells with *SLC25A4* knockdown showed significantly increased proliferation, migration, and invasion, as well as a significant decrease in apoptosis. Here, we explored the transcriptional status, protein levels, functional pathways, mutations, and prognostic associations of *SLC25A4* in OS. It is hoped that these findings may enhance an understanding of the mechanisms associated with OS occurrence and development and help in improving the accuracy of prognosis prediction for OS patients. Hence, the current study may provide a potentially effective target for OS diagnosis and intervention.

The *SLC25* carrier superfamily, the largest solute transport family expressed on the inner mitochondrial membrane (IMM), plays a role in transporting compounds, including amino acids, fatty acids, and nucleotides during metabolism. (Kunji et al., 2020; Ruprecht and Kunji, 2020). Therefore, the *SLC25* carrier superfamily is closely related to various biological pathways including metabolite trafficking, signal transmission, and substance metabolism. (Chen et al., 2022). Several studies have reported the role of *SLC25* in pathophysiological conditions as well as disease progression and showed that it was associated with abnormal tumor metabolism. (Ruprecht and Kunji, 2020). *SLC25A4* encodes a protein also known as adenine nucleotide translocator 1 (ANT1), that participates in ATP/ADP exchange on the IMM. (Klingenberg, 2008; Lu et al., 2017; Zhang et al., 2017). Bertholet *et al* reported that ANT was a key protein required for mitophagy in several cell types and showed that ANT played a novel function as an essential mediator of mitophagy in normal and disease conditions. (Hoshino et al., 2019). Mitochondrial permeability transition pore (mPTP), a structure formed in the IMM, is thought to be the basis for regulating apoptosis, and ANT has been found to be a pore-forming component of mPTP. (Bround et al., 2020). It has been reported that the expression of ANT1 is associated with several apoptotic processes, including caspase activation, mitochondrial membrane potential collapse, phenotypic alteration, DNA degradation, and other features of apoptosis. (Bauer et al., 1999). ANT is a multifunctional protein that plays a key role in several processes, including tumorigenesis, participates in tumor anabolism, controls oxidative phosphorylation and glycolytic homeostasis, and regulates cell death. (Zhao et al., 2021). For instance, Jang *et al* revealed that ANT1 overexpression induced apoptosis in breast cancer cells and strongly suppressed tumor development both *in vitro* and *in vivo*, suggesting that ANT1 might be an effective target for breast cancer treatment. (Jang et al., 2008). Some chemotherapeutic drugs, including lonidamine (LND), can directly or indirectly stimulate ANT and affect the opening of mPTP, thereby inducing tumor cell death. (Decaudin et al., 1998; Abdel-Wahab et al., 2019). LND possesses anti-cancer effects; however, clinical trials showed the presence of significant toxic side effects. (Wang et al., 2023). ANT can regulate energy metabolism by converting its c-state to m-state to complete the ADP/ATP cycle. (Ruprecht et al., 2019). When ANT is in the c-state, ADP is transported from the cytoplasm to the mitochondrial matrix along with the transport of inorganic phosphate (Pi) and H^+^. On the other hand, when ANT is in the m-state, ATP is transported from the mitochondrial matrix to the cytoplasm. (Springett et al., 2017). ANT is a potentially robust target for drug development; however, it should be noted that different subtypes of ANT play different roles in cancer. (Zhang et al., 2018). For example, ANT1 and ANT3 accelerate tumor cell apoptosis, while ANT2 and ANT4 inhibit apoptosis. (Li et al., 2020). Therefore, ANT1 might also be considered a promising target to study novel anticancer agents and might be a novel biomarker for tumor diagnosis and intervention. (Rochette et al., 2020).

Comprehensive bioinformatics analysis showed that the expression of *SLC25A4* was markedly downregulated in both OS tissues and OS cell lines, and the decrease in its profile was closely associated with worse OS patient prognosis. Furthermore, cell function assays revealed that increased expression of *SLC25A4* significantly reduced OS cell proliferation, migration, and invasion, while enhancing apoptosis, indicating that *SLC25A4* was involved in tumorigenesis, tumor progression, and metastasis. Therefore, *SLC25A4* might be a potential key gene for OS and provide a new target for its treatment, thereby contributing to OS diagnosis, treatment, and patient prognosis.

However, the present study has several limitations. First, due to limitations in surgical indications for OS patients and continuous improvements in chemotherapy regimens, the number of samples collected for RNA sequencing was relatively small. Second, the role and function of *SLC25A4* were studied *in vitro*, and *in vivo* investigations involving *SLC25A4* are thus needed. Finally, the specific pathways and regulatory mechanisms associated with *SLC25A4* in OS still require further investigation.

## 5 Conclusion

In conclusion, this study revealed that the expression of *SLC25A4* was significantly downregulated in patients with OS and was strongly associated with prognosis. We also revealed that the prognosis of patients with enhanced *SLC25A4* expression was superior to that of patients with reduced *SLC25A4* expression. Low *SLC25A4* expression significantly promoted OS cell proliferation, migration, and invasion, and inhibited apoptosis, thus participating in OS development. The study suggested that *SLC25A4* might be a therapeutic target for predicting OS patient prognosis, and whether it contributes to OS diagnosis, treatment, and prognosis still requires further investigation.

## Data Availability

Publicly available datasets were analyzed in this study. This data can be found here: STRING database (https://cn.string-db.org/), TCGA database (https://www.cancer.gov/ccg/research/genome-sequencing/tcga), EGA database (https://ega-archive.org/?lang=zh), HPA database (https://www.proteinatlas.org/), cBioPortal for Cancer Genomics (http://cbioportal.org), GEO database (https://www.ncbi.nlm.nih.gov/geo/), accession number GSE238110. The raw data supporting the conclusions of this article will be made available by the authors, without undue reservation.
