# Supplementary material for: Comprehensive transcriptomic analysis identifies SLC25A4 as a key predictor of prognosis in osteosarcoma
Source: Front Genet. 2024 Jun 18;15:1410145. doi: 10.3389/fgene.2024.1410145 (PMC11217516; doi:10.3389/fgene.2024.1410145)
Supplement: Supplementary file 1 [file DataSheet1.zip › Supplementary Material Presentation/Figure_legends SuppInfo.docx]

**Figure_legends SuppInfo**

**Figure S1.** Heatmap of the distribution of DEGs in OS tissues compared to normal tissues.

**Figure S2.** Heatmap of the distribution of DEGs in OS tissues compared to normal tissues. DEGs, differentially expressed genes.

**Figure S3.** Overall survival of DEGs using Kaplan-Meier plotter. Changes in ACTN2, FLNC, HSPB7, LMOD2, MYBPH, MYH7, MYH7B, MYL6B, MYLPF, MYO18B, MYOM2, MYOZ1, MYOZ3, NRAP, SMYD1, SRL, TMOD4, TNNC1, TTN, TXLNB, and UNC45B genes were not correlated with overall survival of OS patients.

**Figure S4.** Expression levels of *SLC25A4, CASQ1, CASQ2*. (A) mRNA expression levels of *CASQ1, CASQ2*, and *SLC25A4* were verified using the TCGA database. (B) protein expression level of *CASQ1* was verified using Western blot. (C) protein expression level of *CASQ2* was verified using Western blot.
