# Supplementary material for: Comprehensive transcriptomic analysis identifies SLC25A4 as a key predictor of prognosis in osteosarcoma
Source: Front Genet. 2024 Jun 18;15:1410145. doi: 10.3389/fgene.2024.1410145 (PMC11217516; doi:10.3389/fgene.2024.1410145)
Supplement: Supplementary file 1 [file DataSheet1.zip › Supplementary Material Presentation/Table_S1.docx]

**Table S1** Functional roles of 10 hub genes

| Gene symbol | Full name | Function |
| --- | --- | --- |
| DES | Desmin | Structural constituent of cytoskeleton, related to [striated muscle contraction pathway](https://pathcards.genecards.org/card/striated_muscle_contraction_pathway) and [Cardiac conduction](https://pathcards.genecards.org/card/cardiac_conduction) |
| CASQ1 | Calsequestrin 1 | calcium regulator in the mitochondria of skeletal muscle, which functions as a luminal sarcoplasmic reticulum calcium sensor in both cardiac and skeletal muscle cells |
| CASQ2 | Calsequestrin 2 | calcium binding protein that stores calcium for muscle function, related to catecholaminergic polymorphic ventricular tachycardia 2 (CPVT2) |
| KLHL31 | Kelch Like Family Member 31 | Transcriptional repressor in MAPK/JNK signaling pathway, Involved in negative regulation of JNK cascade and negative regulation of protein phosphorylation |
| MYBPC2 | Myosin Binding Protein C2 | Thick filament-associated protein which modifies the activity of actin-activated myosin ATPase |
| SYNPO2L | Synaptopodin 2 Like | Actin-associated protein which is predicted to be involved in positive regulation of Rho protein signal transduction, and positive regulation of stress fiber assembly |
| TNNT1 | Troponin T1 | Tropomyosin-binding subunit of troponin, which confers calcium-sensitivity to striated muscle actomyosin ATPase activity |
| PDLIM3 | PDZ And LIM Domain 3 | involved in cytoskeletal assembly, and related to [Invasive Bladder Transitional Cell Carcinoma](https://www.malacards.org/card/invasive_bladder_transitional_cell_carcinoma) and [Facioscapulohumeral Muscular Dystrophy 1](https://www.malacards.org/card/facioscapulohumeral_muscular_dystrophy_1) |
| SLC25A4 | Solute Carrier Family 25 Member 4 | ADP:ATP antiporter which regulates mitochondrial energy output by maintaining a delicate balance between ATP production and thermogenesis and promotes mitophagy via interaction with TIMM44. |
| MYL3 | Myosin Light Chain 3 | Regulatory light chain of myosin, related to calcium ion binding and structural constituent of muscle |
