# Supplementary figures and images for: Comprehensive transcriptomic analysis identifies SLC25A4 as a key predictor of prognosis in osteosarcoma
Source: Front Genet. 2024 Jun 18;15:1410145. doi: 10.3389/fgene.2024.1410145 (PMC11217516; doi:10.3389/fgene.2024.1410145)

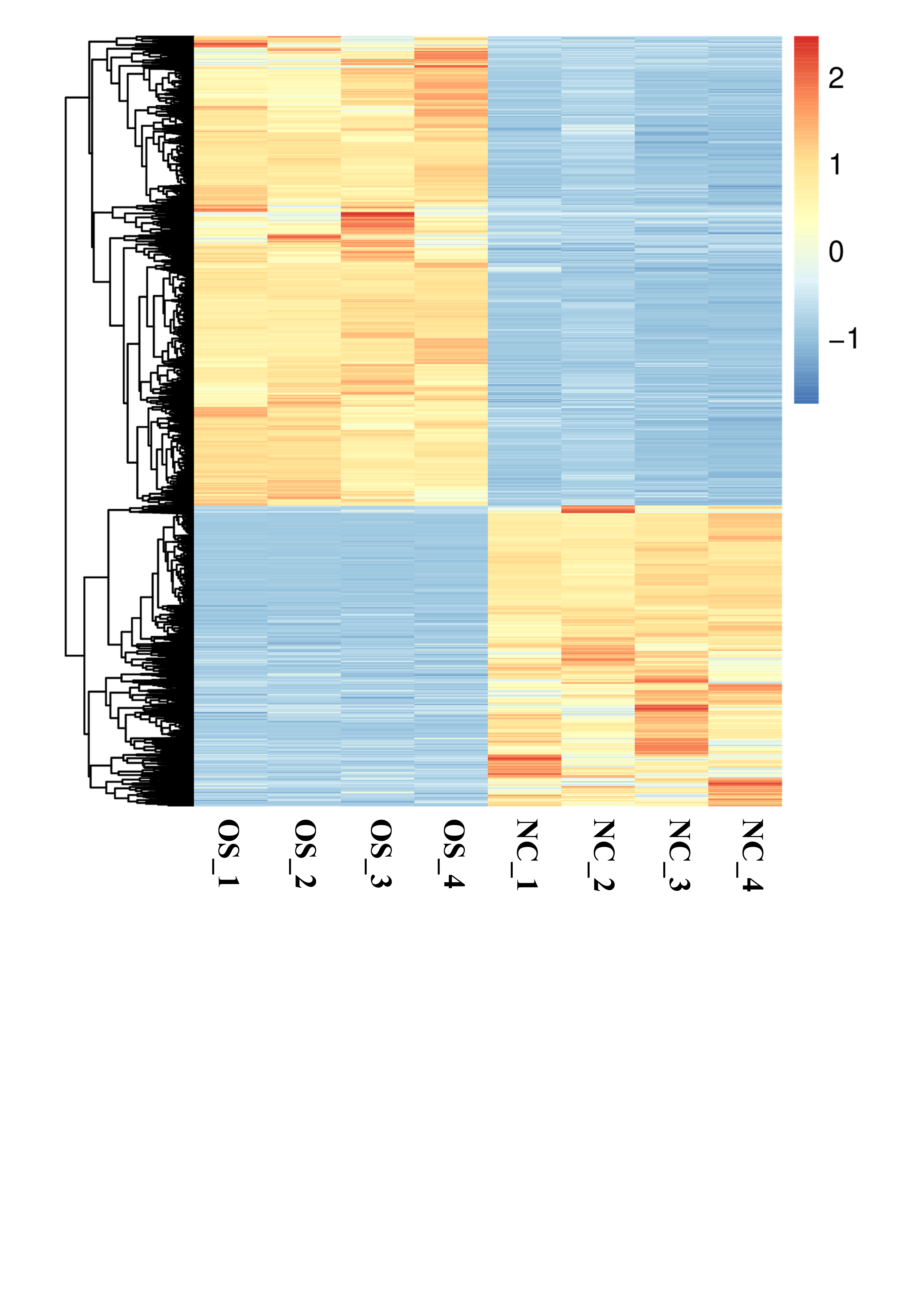

Supplement: Supplementary file 1 [file DataSheet1.zip › Supplementary Material Presentation/Figure S1.TIF]

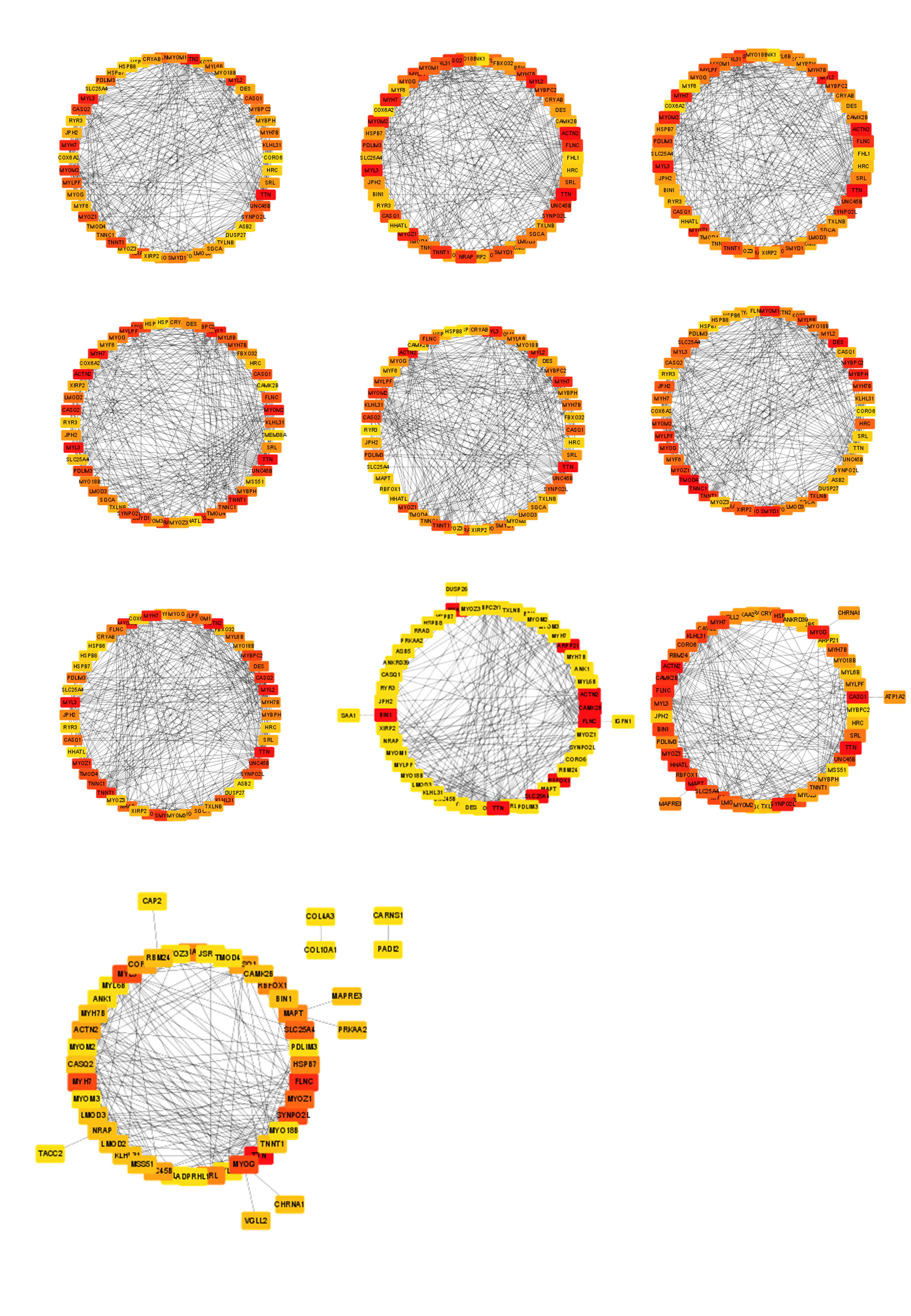

Supplement: Supplementary file 1 [file DataSheet1.zip › Supplementary Material Presentation/Figure S2.TIF]

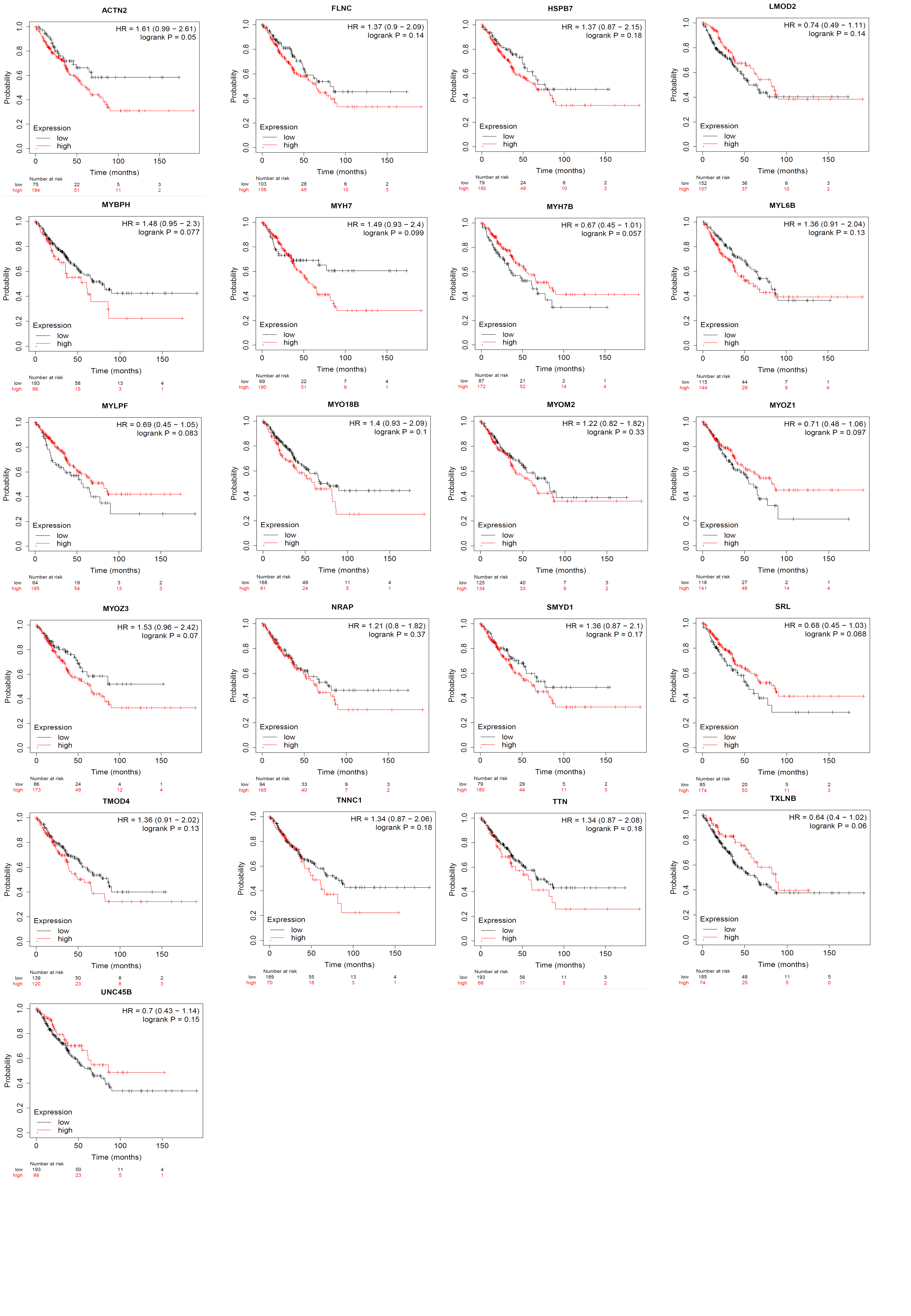

Supplement: Supplementary file 1 [file DataSheet1.zip › Supplementary Material Presentation/Figure S3.TIF]

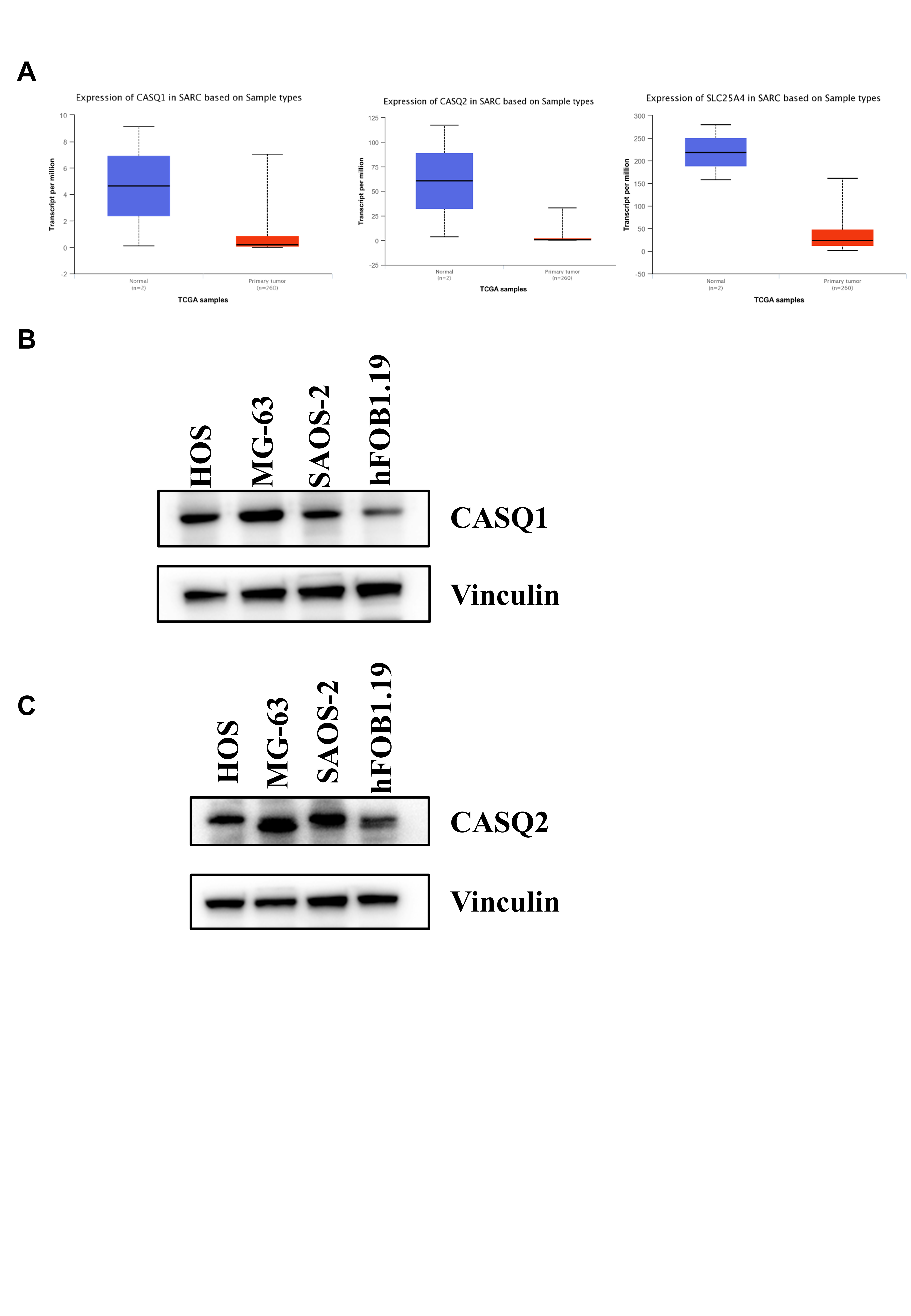

Supplement: Supplementary file 1 [file DataSheet1.zip › Supplementary Material Presentation/Figure S4.TIF]

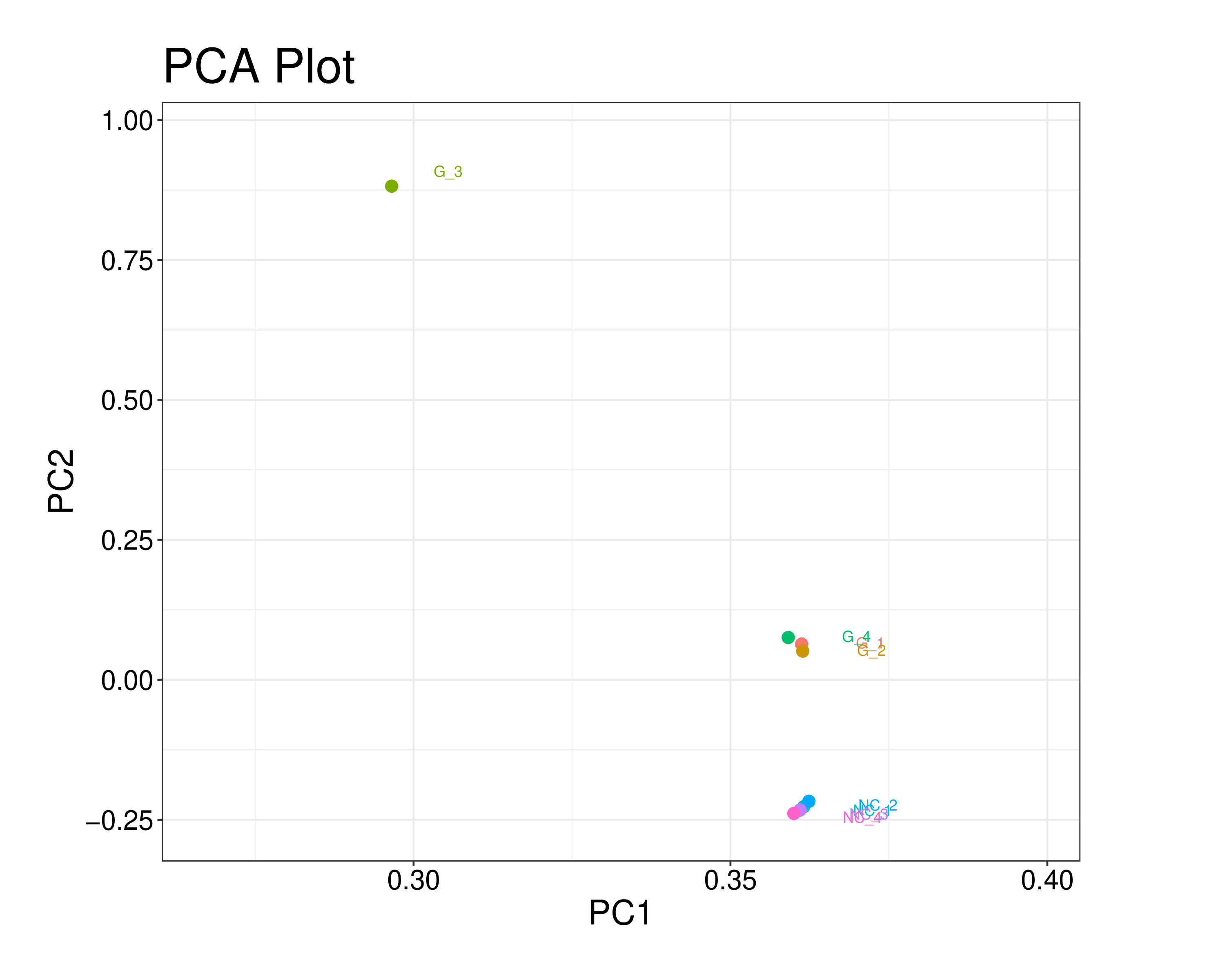

Supplement: Supplementary file 2 [file Image1.TIF]
